# The Microbiome in Connective Tissue Diseases and Vasculitides: An Updated Narrative Review

**DOI:** 10.1155/2017/6836498

**Published:** 2017-08-01

**Authors:** Rossella Talotta, Fabiola Atzeni, Maria Chiara Ditto, Maria Chiara Gerardi, Piercarlo Sarzi-Puttini

**Affiliations:** ^1^Department of Rheumatology, ASST Fatebenefratelli-Sacco, Milan, Italy; ^2^IRCCS Galeazzi Orthopedic Institute, Milan, Italy

## Abstract

**Objective:**

To provide a narrative review of the most recent data concerning the involvement of the microbiome in the pathogenesis of connective tissue diseases (CTDs) and vasculitides.

**Methods:**

The PubMed database was searched for articles using combinations of words or terms that included systemic lupus erythematosus, systemic sclerosis, autoimmune myositis, Sjögren's syndrome, undifferentiated and mixed CTD, vasculitis, microbiota, microbiome, and dysbiosis. Papers from the reference lists of the articles and book chapters were reviewed, and relevant publications were identified. Abstracts and articles written in languages other than English were excluded.

**Results:**

We found some evidence that dysbiosis participates in the pathogenesis of systemic lupus erythematosus, systemic sclerosis, Sjögren's syndrome, and Behçet's disease, but there are still few data concerning the role of dysbiosis in other CTDs or vasculitides.

**Conclusions:**

Numerous studies suggest that alterations in human microbiota may be involved in the pathogenesis of inflammatory arthritides as a result of the aberrant activation of the innate and adaptive immune responses. Only a few studies have explored the involvement of dysbiosis in other CTDs or vasculitides, and further research is needed.

## 1. Introduction

The human microbiota harboured by each person consists of 10–100 trillion symbiotic microbial cells, mainly bacteria in the gut, but also viruses, yeasts, protozoa, and even helminths. The sum of human microbes and their genes existing within and on the human body (collectively known as the microbiome) has been found to be a principal factor in human health and disease [1]. Humans and microbes have coevolved to establish a symbiotic relationship over time, but perturbations, known also as dysbiosis, may occur and drive several diseases, including autoimmune disorders. Over the last few decades, new insights provided by DNA sequence-based analyses of human microbial communities have renewed interest in mucosal immunology and suggest that alterations in the human microbiome can also affect the development of rheumatic diseases.

The concept that human microbiota may modulate systemic autoimmunity is not new, but the underlying mechanisms of autoimmune regulation by the microbiome are just beginning to emerge [2]. Studies of animal models published 30 years ago demonstrated a relationship between the development of inflammatory arthritis and the presence/absence of some intestinal bacteria [3, 4], and, more recently, many studies have drawn attention to the potential role of the oral microrganism *Porphyromonas gingivalis* in the development of rheumatoid arthritis (RA) [5]. A recent study of the lung microbiome in a cohort of patients with early RA has found distal airway dysbiosis similar to that detected in sarcoidol lung inflammation [6], but, although various studies have investigated the different composition of gut microbiota in patients affected by RA and spondyloarthritis (SpA), the complex mechanisms by which microbes influence the pathogenesis of autoimmune diseases are still unknown.

Connective tissue diseases (CTDs) encompass a wide group of immune-mediated diseases, characterized by the inflammation of the connective tissues of the body sustained by the activation of the immune system against self-epitopes expressed on cells and matrix. Vasculitides share similar pathogenetic mechanisms and are characterized by an immune-mediated response against components of the vascular tree. Genetic predisposition is necessary but not sufficient to give raise to these diseases, and an environmental trigger, including infections or changes in microbiome composition, is thought to be required for the onset. Given the spread of connective tissues all over the body, clinical manifestations of CTDs are polyhedral and may involve skin, joints, and visceral organs. Similarly, vasculitides have a wide range of clinical manifestations, according to the vascular district involved by the inflammatory process.

Like in inflammatory arthritides, it is presumable that, in genetic predisposed individuals, dysbiosis activates several immune pathways favouring in turn the development of CTDs or vasculitides.

As there are currently only limited animal and human data available concerning the potential link between the microbiome and systemic autoimmune diseases other than SpA and RA, the aim of this narrative review is to provide an updated view of the involvement of microbiome in the pathogenesis of CTDs and vasculitides.

## 2. Materials and Methods

### 2.1. Sources and Selection Criteria

The PubMed database was searched for articles using combinations of words or terms that included connective tissue diseases (systemic lupus erythematosus, systemic sclerosis, autoimmune myositis, Sjögren's syndrome, undifferentiated and mixed connective tissue disease, and vasculitis), microbiota, microbiome, and dysbiosis. Papers from the reference lists of the articles and book chapters were reviewed, and relevant publications were identified. Abstracts and articles written in languages other than English were excluded.

## 3. Results

We selected 55 articles concerning the role of the microbiome in CTDs and vasculitides; the findings are described below, divided by the type of rheumatic disease.

### 3.1. Systemic Lupus Erythematosus

Systemic lupus erythematosus (SLE), the most emblematic CTD, mainly affects women of childbearing age. As in the case of other autoimmune diseases, it has been hypothesized that an infectious stimulus triggers the onset of SLE by inducing chronic immune system activation [7]. No specific infective agent has yet been isolated, but various candidates have been proposed (including *Epstein-Barr virus*, *cytomegalovirus*, retroviruses, and *human parvovirus B19*), and there is some concern about the possible role of unbalanced microbial microbiota and the prevalence of some pathogens in the gut and mucosa of SLE patients [8].

Up to 50–80% of SLE patients can suffer from skin and mucosal manifestations, in which changes in mucocutaneous commensal flora composition can lead to the proliferation of pathogen species driving chronic inflammation [9]. The innate immunity is activated as the first step during infections sustained by intracellular pathogens, and the secretion of cytokines like type I interferons, which include IFN-*α* and IFN-*β*, is required to modulate the immune response leading to cell apoptosis and pathogen clearance. In SLE, type I interferons are typically overexpressed and associated to disease activity and can induce the hyperactivation of myeloid dendritic cells activating in turn autoreactive T lymphocytes that play a role in SLE pathogenesis [10].

In a study of 84 SLE patients, Conti et al. detected *Staphylococcus aureus* colonising the nasal mucosa and, although the rate of occurrence was similar to that observed in healthy controls, this may be associated with a distinct SLE phenotype characterized by renal and skin involvement and a higher likelihood of anti-dsDNA, anti-Sm, anti-SSA, anti-SSB, and anti-RNP antibody positivity [11]. The activation of cells belonging to the innate immune system via the interactions of pathogen-associated molecular patterns (PAMPs) with Toll-like receptors (TLRs) or the nucleotide-binding oligomerization domain-containing protein 2 (NOD2) cascade, along with the subsequent production of IFNs, plays a key role in the clearance of infections sustained by *Staphylococci,* although it may contribute at the same time to the virulence of some strains of these microorganisms, like strain 502A [12]. However, the authors did not distinguish among different strains of *Staphylococcus aureus* colonising nasal mucosa of SLE patients, which may account for different burdens of cytokine production and different pathogenetic pathways, but only described the association between bacteria and clinical and laboratory alterations.

Periodontal inflammation has also been associated with SLE. In a study of 52 SLE patients and 52 controls, Corrêa et al. found that the risk of periodontitis sustained by *Fretibacterium*, *Prevotella nigrescens*, and *Selenomonas* spp. was higher in patients than in controls and was related to the local release of interleukin- (IL-) 6, IL-17, and IL-33 [13]. The hyperexpression of C3 has been associated with the maintenance of periodontitis sustained by *Porphyromonas gingivalis* in experimental animal models. According to Maekawa et al., neutralising C3 by adding the compstatin analogue Cp40 may prevent local inflammation and the subsequent release of IL-17 or receptor activator of nuclear factor kappa-B ligand (RANKL), perhaps by restoring dysbiosis [14]. Immune complexes formed by ribonucleoproteins, autologous nucleic acids, and immunoglobulin G (IgG) bind to fragment cristallizable receptors (FcgRIIa) and trigger intracellular responses by binding endosomal TLRs, thus inducing complement activation and IFN production. C3 is a crucial molecule in generating IFN signature in SLE, and C3-deficient mutant mice have less type I IFN-related apoptosis and cytokine production [15]. Although the role of complement fractions in favouring or counteracting the growth of pathogenic species in the gingival mucosa of this population of patients is still unknown, the findings suggest that gingival inflammation may represent a chronic stimulus for the local activation of innate immune cells and induce complement consumption and IFN signature generation.

A number of SLE studies have investigated the composition of gut bacteria in animal models and humans and have been recently addressed in a review by Neuman and Koren [16]. In rare cases, SLE patients may suffer from noninfectious SLE enteritis, which is usually sustained by a visceral or serosal vasculitis of the bowel. Gut dysbiosis could be at the basis of local or systemic inflammation, by modulating the immune response [17]. Engagement of TLR9 by commensal flora may modulate the T-effector/regulator ratio in nonautoimmune animal models and lead IFN signature in SLE. However, SLE enteritis and the unbalance in gut flora composition are still unclear.

Zhang et al. found a gender-dependent alteration in the composition of gut microbiota in stool and colon samples taken from lupus-prone MRL/Mp-Faslpr (MRL/lpr) mice: female mice had higher levels of Lachnospiraceae and Bacteroidetes S24-7 and lower levels of *Bifidobacterium* and Erysiopelotrichaceae, with no significant difference in Lactobacillaceae, although the levels of the latter family (and Firmicutes phylum) were significantly lower in the MRL/lpr mice than in controls and were restored after treatment with oral retinoic acid [18]. Hevia et al. evaluated the intestinal microbial composition of 20 SLE patients and 20 matched controls by analysing fecal samples using Torrent 16S rRNA gene-based sequencing [19] and found a significant reduction in the Firmicutes/Bacteroidetes phyla ratio in the patients, which may account for the glycan degradation, lipopolysaccharide biosynthesis, and oxidative phosphorylation described in SLE patients. Similar results were also obtained in a Chinese SLE study cohort, in which the authors also isolated nine genera (*Rhodococcus, Eggerthella, Klebsiella, Prevotella, Eubacterium, Flavonifractor*, and Incertae sedis) that were significantly increased in comparison with controls [20]. According to Rodríguez-Carrio et al., the altered Firmicutes/Bacteroidetes ratio in the gut of SLE patients may account for the hyperproduction of free fatty acids (FFAs) that are associated with impaired endothelial dysfunction characterized by increased levels of leptin, interferon gamma-inducible protein-10, epidermal growth factor, IL-8, and monocyte chemoattractant protein-1 [21]. Lopez et al. have shown that altered gut microbial composition may affect the final differentiation of T helper (Th) subsets in SLE patients in an *in vitro* study of 37 SLE patients and 36 controls: they showed that an imbalance of Firmicutes and Bacteroidetes in fecal samples was associated with Th17 responses whereas enrichment with *Bifidobacterium bifidum LMG13195* or a mixture of two clostridium strains, *Ruminococcus obeum DSM25238* and *Blautia coccoides DSM935*, restored the number of T regulatory cells, thus providing a rationale for the use of probiotic therapy [22]. In another study, Rojo et al. found significant differences in the metabolite landscape but not in the microbial composition in SLE patients versus healthy controls. SLE patients had reduced levels of homoserine lactone and N-acetylmuramic acid and increased level of ribose-1,5-bisphosphate, which, in contrast to healthy subjects, were not influenced by body mass index (BMI) [23]. Furthermore, studies on gut dysbiosis in SLE demonstrated a reduced ratio of Firmicutes/Bacteroidetes that may be at the basis of an altered differentiation of Th effectors, selecting Th17 clones to the detriment of T regulator ones, with the subsequent production of proinflammatory cytokines. The use of prebiotics or probiotics in these patients seems promising; however, randomised controlled trials (RCTs) are still unavailable.

Some experiments in animal models have shown that caloric restriction, as well as the assumption of polyunsaturated fatty acids, vitamins A, D, and E, and phytoestrogens may ameliorate SLE disease activity [24]. Dietary changes may also affect intestinal virome thus preventing cytotoxic and proapoptotic responses aiming to eradicate intracellular microorganisms. Cuervo et al. reported a significant association between the intake of flavone, flavanones, dihydrochalcones, and polyphenols and the growth of *Blautia*, *Lactobacillus*, and *Bifidobacterium* in a cohort of 20 SLE female patients [25]. In another experiment on lupus-prone SNF_1_ mice, the administration of acidic water restrained the course of glomerulonephritis and the production of cytokines and autoantibodies, inducing meantime a restore in the gut microbial flora (higher *Lactobacillus reuteri* and *Turicibacter* spp. colonisation in acidic water-drinking mice) [26]. Similar results have been reported with the use of *Lactobacilli* GMNL-32, GMNL-89, and GMNL-263 on the course of SLE hepatitis in lupus-prone mice [27].

To summarise, there are emerging evidences that dysbiosis represents one rawplug inside the complex pathogenesis of SLE; however, given the modest knowledge on the beneficial effects of probiotics and prebiotics and the lack of RCTs, no clear therapeutic strategies can be currently drawn. Additionally, the use of immunomodulators and immunosuppressive drugs may alter the composition of commensal flora, selecting pathogens able to chronically stimulate the immune system.

### 3.2. Systemic Sclerosis

Systemic sclerosis (SSc) is a connective tissue disease that may affect the entire gastrointestinal (GI) tract in up to 90% patients [28], who often have oral telangiectasias, esophageal dysmotility, small intestinal bacterial overgrowth, or delayed intestinal transit. The most representative characteristic of GI involvement is fibrosis of the muscular tunic, which is associated with reduced peristalsis [29, 30]. However, there is some evidence that there are mucosal alterations associated with altered intestinal permeability and possibly dysbiosis. Increased intestinal permeability would favour the translocation of bacterial products from the lumen to the submucosal layer, thus leading to aberrant activation of the local innate and adaptive immune system [31–33].

Several studies have demonstrated that vascular damage occurs throughout the intestinal tract and that mucosal abnormalities (including watermelon stomach, intestinal telangiectasias, and angiodysplasia) may be present in more than 50% of SSc patients and are usually associated with a more pronounced vasculopathic phenotype (i.e., digital ulcers and pulmonary artery hypertension) [34]. Together with endothelial dysfunction, defective control of microvascular tone may be responsible for tissue hypooxygenation and the production of reactive oxygen species (ROS), thus leading to chronically defective enterocytes, which may lose their ability to counteract the proliferation of pathogenic microorganisms.

Only a few published studies have so far investigated the role of dysbiosis in SSc.

Andréasson et al. used a genome-based microbiota test of stool samples to study the gut flora composition of 98 SSc patients [35] and found that 75.5% had dysbiosis (low levels of *Faecalibacterium prausnitzii* and/or Clostridiaceae) and that this was significantly associated with esophageal dysmotility, micronutrient deficiency, skin telangiectasias, pitting scars, pulmonary fibrosis, and high serum levels of inflammation markers. Volkmann et al. examined by means of Illumina HiSeq 2000 16S sequencing of cecum and sigmoid mucosal lavage samples taken from 17 SSc patients and a healthy control group and found decreased levels of commensal bacteria such as *Faecalibacterium* and *Clostridium* and increased levels of pathogenic bacteria such as *Fusobacterium* and *g-Proteobacteria* in the patients, who also had increased levels of *Bifidobacterium* and *Lactobacillus* and a reduced *Bacteroides fragilis/Fusobacterium* ratio, especially in the case of the most severe GI symptoms [36].

Measurement of fecal calprotectin is a noninvasive tool for monitoring bowel inflammation and may also give indirect information on the innate immune cells' activation following a microbial insult. The molecule, formed by the dimerization of the proteins S100A8 and S100A9 and mainly expressed in the cytosol of polymorphonuclear cells, prevents the infections sustained by virulent strains of bacteria or fungi, but its concentration is also arisen in autoimmune inflammatory bowel diseases (IBDs). Marie et al. detected increased levels of fecal calprotectin (>50 *μ*g/g) in 93 of their 125 SSc patients and found that fecal calprotectin concentration significantly correlated with intestinal motor dysfunction and small intestinal bacterial overgrowth as assessed by means of glucose H_2_/CH_4_ breath testing [37].

Changes in the lung and skin microbiome of SSc patients may also occur. Polyhedral cutaneous and pulmonary manifestations, including puffy fingers, skin and lung fibrosis, and alveolitis, led to the discovery of four genotypes that may lead to a more pronounced fibrotic or inflammatory phenotype [38]. The inflammatory phenotype is characterized by the upregulation of genes involved in the autoimmune and autoinflammatory cascades triggered by the stimulation of TLRs, converging to activate the nuclear factor kappa-light-chain-enhancer of activated B cells (NFkB) and then activating a complex cascade involving Th2 lymphocytes, M2 macrophages, Th17 cells, and fibroblasts [39].

Many microbial products have been postulated as inducing the activation of lung and skin macrophages and dendritic cells through TLRs. Fungi are commensal components of the microbiome of the skin and lung, and the lung mycobiome of healthy subjects is mainly represented by *Aspergillus* spp., whereas *Candida* spp. become prevalent under pathological conditions (including idiopathic pulmonary fibrosis and interstitial lung disease) [40]. However, no study has yet evaluated microbiome or mycobiome composition in the lungs of SSc patients. One study used ribosomal RNA sequencing of forearm skin biopsies taken from patients with early (<6 months) diffused and limited SSc and healthy controls and found the increased expression of *Rhodotorula glutinis* sequences in the patient samples, but no significant differences in bacterial or viral characterisation [41]. It can be argued that *Rhodotorula* can cause SSc skin fibrosis as it may activate the innate immune system and induce lung granulomatous diseases and peritoneal fibrosis, but there is a lack of studies characterising the mycobiome of unaffected skin sites and those affected by disease.

In brief, similarly to SLE, there is some evidence that SSc patients may also suffer from a multidistrict imbalance of commensal and pathogenic microorganisms and antibiotic or probiotic treatments may have some beneficial effects [42, 43]. However, it is still unknown where the primitive dysbiotic insult occurs (skin or visceral organs), which organ may be firstly affected, and whether dysbiosis could be the consequence rather than the cause of the anatomic modifications affecting connective tissues.

### 3.3. Sjögren's Syndrome

Sjögren's syndrome (SS) is a chronic inflammatory autoimmune disease involving the exocrine glands (mainly the lacrimal and salivary glands) that has extraglandular systemic repercussions in 71% of cases [44]. Glandular tissue inflammation characterized by the infiltration of dendritic cells, CD4+ T lymphocytes, and B cells leads to the final destruction of the acini and reduces the secretion of fluids containing antimicrobial factors, and the subsequent disruption of the skin and mucosal barrier may lead to dysbiosis and a higher risk of colonisation by pathogenic species.

However, in a study from Lugonja et al., the authors did not find any increase in the prevalence of periodontitis in 39 SS patients in comparison with 36 RA and 23 osteoarthritis controls on the basis of a search for specific serum antibodies against ten oral/periodontal bacteria [45].

It has been hypothesized that dysbiosis may condition the activation of the immune system and influence the severity of SS. For example, it has been demonstrated that postnatal gut colonisation by different *Bifidobacterium species* may influence the response of the immune system to microbial stimulation by modulating the production of salivary secretory IgA and that *Bacteroides fragilis* colonisation is associated with a less intense production of cytokines and chemokines by immune cells following stimulation with lipopolysaccharide (LPS) [46]. de Paiva et al. used animal models and humans to demonstrate that, in comparison with controls, there was significant dysbiosis in the stools of SS model mice (with a prevalence of *Enterobacter*, *Escherichia/Shigella*, and *Pseudomonas*) and that histological changes in their mucosae (monocyte infiltration, a reduction in the number of goblet cells, and barrier disruption) were more pronounced after 10 days of exposure to antibiotics [47]. However, they did not find any significant difference in the overall microbial composition of the conjunctiva and tongue between SS patients and healthy controls, although there was a prevalence of *Streptococcus* and a decrease in *Leptotrichia* and *Fusobacterium* levels in the SS tongue samples. The fecal microbiota of the SS patients contained a higher proportion of *Pseudobutyrivibrio*, *Escherichia/Shigella*, *Blautia*, and *Streptococcus* and fewer *Bacteroides*, *Parabacteroides*, *Faecalibacterium*, and *Prevotella*. Interestingly, the severity of ocular (but not systemic) symptoms assessed using the unweighted 12-domain European League Against Rheumatism (EULAR) Sjögren's syndrome disease activity index (ESSDAI) was significantly related to gut flora diversity.

Szymula et al. investigated whether the production of autoantibodies against RoSSA, which represent a distinctive trait and classification criterion for SS [48], may be triggered by the recognition of a cross-reactive bacterial peptide by the adaptive immune system [49]. Using Ro60 reactive T cell hybridomas from HLA-DR3 transgenic mice, they demonstrated that a cross-reactive peptide from the von Willebrand factor type A domain protein (vWFA) produced by *Capnocytphaga ochracea* was a potent activator of T cells. *C. ochracea* is a gram-negative, anaerobic bacterium that is usually isolated in gingival sites and dental plaques, and so the disrupted mucosal barrier in the oral cavity or gut of SS patients may underlie the proliferation of pathogens capable of chronically activating the immune system, possibly by means of a mechanism of molecular mimicry.

In conclusion, the altered functioning of the mucosal and cutaneous barrier observed in SS may favour the colonisation of microbial pathogen species, which may further activate the immune response by molecular mimicry or by a direct interaction with TLRs on local dendritic cells. However, given the paucity of scientific evidence, the exact role of the microbiome in SS is still unclear.

### 3.4. Idiopathic Inflammatory Myopathies

No data are currently available concerning the microbial composition or pathogenic role of the microbiome in autoimmune myositides. Idiopathic autoimmune myopathies include dermatomyositis, polymyositis, necrotising autoimmune myositis, and sporadic inclusion-body myositis. Each form affects skeletal muscle and may overlap with other CTDs, but inflammatory myopathies differ in terms of their clinical aspects, histological findings, and myositis-specific antibodies [50].

There is very little evidence supporting a pathogenic role of dysbiosis in the onset of inflammatory myopathies. A review by Bleau et al. discussed the effect of a high-fat diet on intestinal microbial composition and its further repercussions on adipose tissue and skeletal muscles [51]. A high intake of saturated fatty acids may select the survival of LPS-bearing bacterial species, including Enterobacteriaceae, at the expense of Bacteroidetes, thus inducing the TLR activation of resident macrophages in the gut, adipose tissue, and skeletal muscles. Saturated fatty acids per se may also trigger TLR2 and TLR4 signalling, which may pave the way for chronic proinflammation and the consequent release of tumour necrosis factor-alpha (TNF-*α*), IL-6, and chemokines that may contribute to decreasing protein synthesis, oxidative stress, and insulin-resistance in skeletal muscle tissue. However, despite this favourable pathogenic pathway and some evidence indicating the potential development of inflammatory myopathies after infections or vaccinations [52], no study has yet investigated the differences in microbial taxonomic composition in autoimmune myopathies.

### 3.5. Other Connective Tissue Diseases

There are no available data concerning the role of the microbiome in undifferentiated CTDs (UCTDs), mixed CDT (MCTD), relapsing polychondritis, or other overlap syndromes.

### 3.6. Vasculitides

The vasculitides are a wide spectrum of diseases characterized by chronic inflammation of small, medium-sized, and large vessels. Healthy vessels may harbour their own commensal microbiome, but colonisation of the vascular tree by pathogenic microbial agents, including *Chlamydia pneumoniae*, *Porphyromonas gingivalis*, *Aggregatibacter actinomycetemcomitans*, *Staphylococcus*, and *Stenotrophomonas* spp., has recently been described in a number of vascular diseases, including atherosclerosis and aneurysms [53]. Although little evidence is currently available, it can be presumed that vascular dysbiosis may account for the chronic inflammation occurring in systemic vasculitides.

Giant cell arteritis (GCA) is a systemic granulomatous vasculitis affecting large vessels that mainly occurs in the elderly and has a typical cyclic pattern every 5–7 years. It has been postulated that an infectious stimulus may underlie its onset, but the etiological agent is still unknown. In a DNA sequencing study of the temporal artery biopsies of 17 GCA patients and five controls, Bhatt et al. did not find any distinctive microbiome signature in the patients and the isolation of *Propionibacterium acnes*, *Escherichia coli*, and *Moraxella catarrhalis* in both GCA and healthy samples mainly reflected external contamination [54].

Kinumaki et al. made a longitudinal metagenomic analysis to assess intestinal microbial variations in a cohort of 28 patients with Kawasaki disease (KD) [55], a form of vasculitis affecting the medium-sized vessels of mainly Asiatic children aged 6–11 months. It is usually associated with febrile spikes, lymphadenopathy, and mucosal and skin manifestations, which suggest an infectious etiopathogenesis although its cause is unknown. The authors found that five streptococcal species (*S. pneumonia*, *pseudopneumoniae*, *oralis*, *gordonii*, and *sanguinis*) and, in some cases, the genera *Rothia* and *Staphylococcus* were the most abundantly represented microorganisms in the intestinal tract of KD patients, and their prevalence increased during the phases of disease reactivation. On the contrary, the presence *Ruminococcus*, *Blautia*, *Faecalibacterium*, and *Roseburia bacteria* increased during disease remission.

There are no published data concerning dysbiosis in small vessel vasculitides, although it has been demonstrated that gut dysbiosis in glomerulonephritis mice models (with a prevalence of *Escherichia coli* or *Citrobacter rodentium*) may locally expand Th17 lymphocytes, which could then migrate to the kidney through a chemokine pathway involving C-C motif chemokine ligand 20 (CCL20) and C-C motif chemokine receptor 6 (CCR6) [56]. Accordingly, patients with antineutrophil cytoplasmic antibody- (ANCA-) associated vasculitis develop a form of necrotising glomerulonephritis sustained by the infiltration of Th17 cells that may be preactivated in the gut by a change in microbial composition. Disrupted gut tolerance of microbial agents may also be reflected by the detection of ANCAs in patients with inflammatory bowel diseases such as Crohn's disease, in whom (together with anti-glycoprotein 2, anti-granulocyte macrophage colony-stimulating factor, anti-Saccharomyces cerevisiae antibodies, and other antibody subsets) they represent a valid means of diagnosing and monitoring the course of the disease [57].

A number of studies have investigated the role of dysbiosis in the pathogenesis of Behçet's disease (BD), a variable-vessel vasculitis characterized by recurrent mouth and genital ulcers, ocular inflammation, and skin rashes. Its etiopathogenesis is still unknown, but it shares some of the traits of autoinflammatory diseases. Coit et al. investigated the oral microbiome of 31 BD patients and 15 controls by analysing salivary samples using high-throughput sequencing of the 16S rRNA V4 region [58] and found a reduced prevalence of *Alloprevotella rava* and increased colonisation by *Haemophilus parainfluenzae*, neither of which was significantly affected by concomitant immunosuppressive treatments (cyclosporine A, azathioprine, and prednisone) or genetic predisposition (HLA-B/MICA locus variants), but they were slightly restored by periodontitis treatment. The same authors reported a significant reduction in species diversity in BD patients, as measured by the inverse Simpson index.

Seoudi et al. found differences in the microbial composition of the saliva of 54 BD patients, eight subjects affected by recurrent aphthous stomatitis, and 25 controls [59]. Nonulcerous BD oral cavities were mainly colonised by *Rothia denticariosa*, whereas ulcerous BD oral cavities had higher prevalence of *Streptococcus salivarius* and *Streptococcus sanguinis* than those of the subjects in the two control groups.

Using pyrosequencing of the V3-V4 hypervariable regions of the 16rDNA gene, Consolandi et al. found a significant reduction in the genera *Roseburia* and *Subdoligranulum* in fecal samples of 22 BD patients in comparison with 16 controls [60]. These microorganisms belonging to the Clostridiales order are responsible for the production of short-chain fatty acids (SCFAs) that have anti-inflammatory properties, and, accordingly, biochemical stool analysis revealed lower SCFA levels in the BD patients that positively correlated with the reduced *Roseburia* (but not *Subdoligranulum*) colonisation. Similarly, Shimizu et al. made a metagenomic analysis of stool samples from 12 BD patients and 12 healthy subjects [61] and found a reduced prevalence of *Clostridia* and an increased prevalence of *Actinobacteria* and *Lactobacillus* spp. in the patients, with only minimal effects of concomitant immunosuppressive treatments.

In summary, vasculitides represent a heterogeneous group of immune-mediated diseases sustained by different immunologic pathways and involving different-sized vessels. The role of microbiome in vessel inflammation may be based on microbiome unbalance in genetically predisposed patients. However, because data concerning vascular dysbiosis are still unvailable, it can be hypothesized that inflammation may arise from the attempt to counteract dysbiotic changes occurring in other districts, such as the gut or oral mucosa. Among vasculitides, only BD showed altered microbiome in saliva and stool, but the link between alterated microbiome and vascular inflammation is still unclear.  Table 1 resumes the results of the principal studies evaluating dysbiosis in CTDs and vasculitides.

## 4. Discussion

The role of the microbiome in inducing and worsening autoimmune diseases has been widely investigated over the last years. The commensal and pathogenic microorganisms resident in the skin or mucosa are in mutual balance, protected by the integrity of the skin/mucosal barrier and the active surveillance of the innate and adaptive immune systems. The broad spectrum of CTDs is characterized by profound changes in the anatomical and physiological characteristics of mucosa and skin and therefore provides a predisposing environment for the onset of dysbiosis. Once it has occurred, dysbiosis leads to the release of microbial peptides and other molecules that amplify a state of chronic inflammation as a result of the activation of effector cells belonging to both innate and adaptive immune systems. There is considerable evidence that dysbiosis can trigger autoimmune diseases, including RA and SpA, but less is known about microbial composition in CTDs or vasculitides.

Nevertheless, studies of SLE and SSc have demonstrated an imbalance in the composition of the gut and muco-cutaneous flora. A reduced Firmicutes/Bacteroidetes ratio has been found in the gut of SLE patients and reduced levels of Clostridiaceae in fecal samples of SSc patients. These changes in the microbial composition are at the basis of an increased production of proinflammatory FFAs and a reduction in the amount of anti-inflammatory SCFAs, with a subsequent modulation in the cytokine pattern and in the release of leptin. Moreover, reduced gut colonisation by Firmicutes has been related to the inbalance between Th17 and T regulator lymphocytes.

Chronic infections sustained by pathogen strains, including *Staphylococcus aureus*, may activate the innate immune system via the recognition of PAMPs by TLRs or NOD2, or following opsonization with C3 and immunoglobulins, thus fomenting the production of type I IFNs that represent a peculiar signature in SLE pathogenesis. So far, two studies have associated nasal colonisation by *Staphylococcus aureus* and periodontitis sustained by *Fretibacterium*, *Prevotella nigrescens,* and *Selenomonas* spp. with clinical manifestations and the production of some proinflammatory cytokines in SLE patients.

Alterations in the mycobiome may underlie fibrotic skin and lung diseases as the fibrotic skin areas of SSc patients may be colonised by *Rhodotorula glutinis*, which is known to be involved in fibrosing diseases.

Intestinal dysbiosis has also been demonstrated in SS patients, and, interestingly, gingival colonisation by *Capnocytphaga ochracea* in genetically predisposed subjects may lead to the activation of the adaptive immune system through the cross-reactivity against a von Willebrand factor type A domain protein (vWFA) that is produced by the microorganism and Ro-SSA self-antigens.

The evidence that dysbiosis may be involved in the pathogenesis of myositides and vasculitides is weaker, but some studies of BD patients have found taxonomic variations in the microbial composition of the gut that were not affected by concurrent immunosuppressive therapies. Similarly to SLE and SSc, a dysbiosis consisting in a reduction in Clostridiales has been reported in fecal samples of BD patients, with a subsequent hypoproduction of SCFAs having anti-inflammatory properties.

An isolated study assessing the intestinal microbiome in patients affected by KD found an increased expression of *Streptococci*, *Staphylococci*, and *Rothia* during disease's flares. No studies addressed the role of dysbiosis in the onset of small vessel vasculitides, although some experiments on animal models could support the role of intestinal microbiome in the activation of autoreactive Th17 cells in ANCA-associated vasculitides.

Overall, the contribution of dysbiosis in tuning the immune response has a rationale in CTDs and vasculitides, although there are currently less evidences than in chronic autoimmune arthritides. Moreover, the multifaceted expression of these diseases raises the questions about the primitive district in which dysbiosis would occur. In chronic autoimmune arthritides, changes in gut microbiome have been considered the main source of immune system activation. Since joints are sterile sites, the most accredited hypothesis is that effector cells could be primed and activated following the presentation of microbial peptides in the gut and then migrate into joints where they could give raise to the inflammatory cascade via a mechanism of *molecular mimicry*. Alterations in the composition of the commensal flora harboured in the intestinal tract have been detected in SLE, SSc, and SS; however, dysbiosis occurring at mucosal sites, including the mouth, nose, and lungs as well as in the skin, may represent another trigger in these diseases characterized by a high burden of skin and mucosal inflammation. The impairment of the muco-cutaneous barrier, partly related to chronic inflammation, may further favour the chronicity of this mechanism by which pathogen species could survive and maintain inflammation in already inflammed sites. Several studies on BD, characterized by recurrent mouth aphtosis, have demonstrated an unbalanced flora of the mouth, which was associated also to disease activity. Similarly, the onset of other vasculitides may be related to alterations in vessel microbiome, but studies are lacking.

According to above described data, counteracting dysbiosis by means of prebiotics or probiotics may represent a useful tool in preventing or limiting inflammation in CTDs and vasculitides. Implementation with *Lactobacilli* or their substrates has shown some benefits in experimental studies on SLE human and animal cohorts. Unfortunately, no RCTs on the effectiveness of such strategies are available, and currently published studies are not conclusive due to heterogenicity in methodology. It is likely that a normocaloric diet with a high fiber and vitamin content together with a weight control could help patients in reducing the inflammatory burden and could be considered beside the pharmacologic intervention in the future algorithm for the treatment of these diseases.

## 5. Conclusions

In conclusion, several studies suggest that alterations in human microbiota may be involved in the pathogenesis of CTDs or vasculitides; however, due to the lack in a methodologic standardization, the absence of RCTs, the polyhedral manifestations of the diseases, and concomitant treatments as well as the still scarce amount of research in this field, further studies are needed to better understand the real impact of dysbiosis on the course of these diseases and to conceive preventive or therapeutic strategies to counteract microbiome-driven inflammation.

## Figures and Tables

**Table 1:** Principal studies aiming to evaluate the alterations of microbiota in connective tissue diseases and vasculitides.

Author, year	Country	Models	Disease	Sample type	Technology employed	Implicated microbiota	Reference
Hevia et al., 2014	Spain	Human	SLE	Stool	16S rRNA (Ion Torrent PGM Sequencing, PCR analysis)	↓ Firmicutes/Bacteroidetes ratio in SLE pts than HC	[19]
He et al., 2016	China	Human	SLE	Stool	16S rRNA (Illumina Miseq)	↓ Firmicutes genera *Dialister* and *Pseudobutyrivibrio* ↑ Bacteroidetes *Rhodococcus*, *Eggerthella*, *Klebsiella*, *Prevotella*, *Eubacterium*, *Flavonifractor*, and Incertae sedis	[20]
Corrêa et al., 2017	Brazil and USA	Human	SLE	Subgingival dental plaque samples	V4 region of 16S rRNA (Illumina MiSeq)	Higher bacterial loads and decreased microbial diversity ↑ *Fretibacterium*, *Prevotella nigrescens*, and *Selenomonas*	[13]
Arron et al., 2014	USA	Human	SSc	Skin	Integrated Metagenomic Sequence Analysis, DNA microarrays, and 16S rRNA sequencing (Illumina HiSeq 2000)	No difference in bacterial microbiome between SSc and HC ↑ *Rhodotorula glutinis* in SSc	[41]
Andréasson et al., 2016	Sweden	Human	SSc	Stool	The GA-map™ Dysbiosis Test	↓ *Faecalibacterium* and *Clostridium* in SSc than HC More severe dysbiosis in pts with esophageal dysmotility, skin telangiectasias, pitting scars, pulmonary fibrosis, and elevated serum markers of inflammation	[35]
Volkmann et al., 2016	USA	Human	SSc	Cecum and sigmoid mucosal lavage samples	16S rRNA sequencing (Illumina HiSeq 2000)	↓ *Faecalibacterium* and *Clostridium* ↑ *Fusobacterium*, g-Proteobacteria, *Bifidobacterium* and *Lactobacillus* ↓ *Bacteroides fragilis* ↑ *Fusobacterium* in SSc patients with moderate/severe GI tract symptoms	[36]
de Paiva et al., 2016	USA	Human and mice	SS	Conjunctival samples, tongue samples, stool	Human: Ocular conjunctiva: V1-V3 region of 16S rRNA Tongue mucosa & stool: V4 region of 16S rRNA (454 Sequencing/Illumina Sequencing) Mice: Ready-To-Go™ You-Prime First-Strand kit	Mice stool: ↓ *Blautia*, *Alistipes*, *Lactobacillus*, *Allobaculum*, *Bacteroides*, *Desulfovibrio*, *Intestinimonas*, and *Clostridium* ↑ *Enterobacter*, *Parasutterella*, *Escherichia*/*Shigella*, *Pseudomonas*, and *Staphylococcus* Human stool: ↑ *Pseudobutyrivibrio*, *Escherichia*/*Shigella*, *Blautia*, and *Streptococcus* ↓ *Bacteroides*, *Parabacteroides*, *Faecalibacterium*, *Prevotella* versus HC Tongue samples: ↑ *Streptococcus*, ↓ *Leptotrichia* and *Fusobacterium*, ↓ *Bergeyella*, *Peptococcus*, and *Butyrivibrio* genera	[47]
Seoudi et al., 2015	UK	Human	BD	Saliva	Human oral microbe identification microarray (HOMIM) analysis	↑ *Rothia denticariosa* in BD and RAS ulcer sites ↑ *Streptococcus salivarius* in ulcer sites in BD versus RAS ↑ *Streptococcus sanguinis* in BD ulcer sites versus HC	[59]
Consolandi et al., 2015	Italy	Human	BD	Stool	Pyrosequencing of the V3-V4 hypervariable regions of the 16 rDNA gene and biochemical analysis	↓ *Roseburia* and *Subdoligranulum* than HC	[60]
Coit et al., 2016	Turkey, USA, and Sweden	Human	BD	Saliva	V4 region of 16S rRNA (Illumina Sequencing)	↑ *Haemophilus parainfluenzae* ↓ *Alloprevotella rava* and species in the genus *Leptotrichia*	[58]
Shimizu et al., 2016	Japan	Human	BD	Stool	16S rRNA sequencing (Ion Torrent PGM)	↑ *Bifidobacterium* and *Eggerthella* ↓ *Megamonas* and *Prevotella* genera in BD pts versus HC	[61]
Bhatt et al., 2014	USA	Human	GCA	Temporal artery biopsy specimens	Illumina HiSeq V3 sequencing	↑ *Propionibacterium acnes* and *Escherichia coli* in GCA and HC	[54]
Kinumaki et al., 2015	Japan	Human	KD	Stool	Metagenomic Shotgun Sequencing (Illumina Sequencing)	↑ *Rothia*, *Staphylococcus*, and *Streptococcus* in the acute phase; ↑*Ruminococcus*, *Blautia*, *Faecalibacterium*, and *Roseburia* in the nonacute phase	[55]

SLE: systemic lupus erythematosus; SSc: systemic sclerosis, GI: gastrointestinal; SS: Sjögren's syndrome; BD: Behçet disease; RAS: recurrent aphthous stomatitis; GCA: giant cell arteritis; KD: Kawasaki disease; HC: healthy controls; pts: patients; PGM: personal genome machine; PCR: polymerase chain reaction; rRNA: ribosomal ribonucleic acid.
